# Polarization-Dependent Lateral Optical Force of Subwavelength-Diameter Optical Fibers

**DOI:** 10.3390/mi10100630

**Published:** 2019-09-21

**Authors:** Xiangke Wang, Wanling Wu, Yipeng Lun, Huakang Yu, Qihua Xiong, Zhi-yuan Li

**Affiliations:** 1 School of Physics and Optoelectronics, South China University of Technology, Guangzhou 510641, China; 2 Sino-Singapore International Joint Research Institute, Guangzhou Knowledge City, Guangzhou 510663, China; 3 Division of Physics and Applied Physics, School of Physical and Mathematical Sciences, Nanyang Technological University, Singapore 639798, Singapore

**Keywords:** optical force, subwavelength-diameter optical fiber, circular polarization, elliptical polarization

## Abstract

It is highly desirable to design optical devices with diverse optomechanical functions. Here, we investigate lateral optical force exerted on subwavelength-diameter (SD) optical fibers harnessed by input light modes with different polarizations. It is interesting to find that input light modes of circular or elliptical polarizations would bring about lateral optical force in new directions, which has not been observed in previous studies. By means of finite-difference time-domain (FDTD) simulations, detailed spatial distributions of the asymmetric transverse force density are revealed, meanwhile dependence of optical force on input light polarizations, fiber diameters, and inclination angles of fiber endfaces are all carefully discussed. It is believed that polarization-sensitive reflection, refraction, and diffraction of optical fields occur at the interface, i.e., fiber oblique endfaces, resulting in asymmetrically distributed optical fields and thereafter non-zero transverse optical force. We believe our new findings could be helpful for constructing future steerable optomechanical devices with more flexibility.

## 1. Introduction

It is known that flying photons carry momenta, and exchange of light momenta would give rise to optical force [1]. Optical force is trivial but could become significant under certain circumstances. For example, solar sailing, namely space traveling with propulsion from sunlight, has been a long-time dream of human beings [2]. Solar sail spacecraft usually possess large reflective sails that could capture photons from sunlight and use optical force as propulsion for forward traveling. On the other hand, optical force could be very significant in micro-/nano-scale structures, leading to rapid developments of various multi-functional optomechanical devices [3,4,5,6,7,8,9,10,11,12,13,14,15,16,17,18,19,20,21].

Among numerous optical-force-activated devices, optical waveguides have attracted a lot of attention due to advantages including tight confinement and waveguiding, strong near-field interaction, and feasible accessibility. It is noted that subwavelength-diameter (SD) optical fibers appear to be an ideal platform for investigating optomechanical effects and developing ultrasensitive optomechanical transducers [22,23,24,25,26]. Indeed, extensive studies have been performed based on SD optical fibers [27,28,29]. Much effort has been devoted to the theoretical explanations of lateral deflection of SD optical fibers, while carefully considering many practical factors such as fiber geometries and associated physical effects [30,31,32,33,34,35,36]. Additionally, it was found that oblique endfaces of SD optical fibers gave rise to much stronger lateral optical forces compared with flat endfaces [35]. Therefore, it would be more efficient to develop optical force actuated devices based on SD fibers with oblique endfaces.

Aside from practical factors of SD silica optical fibers investigated previously, polarizations of photons can also play an important role in harnessing optical forces. This point is easy to understand while considering the fiber endface, an interface that terminates the optical fiber; all the photon-associated reflection, refraction, and diffraction are polarization-sensitive. It was also found that upon quasi-linear polarized inputs, lateral optical force can only be found in one direction [35], preventing the possibility of SD fiber actuation in multi-dimensions. Therefore, it is desirable to further develop new mechanisms and explore the polarization-dependent optical force exerted on the fiber endface, so as to extend the content of fiber-based optomechanical transducers.

In this paper, we investigated optical force exerted on an SD silica fiber harnessed by input light with different polarizations. It was found that input light of circular or elliptical polarizations would bring about lateral optical force in new directions for the first time. With the help of the numerical three-dimensional finite-difference time-domain (3D-FDTD) method, detailed optical force density distributions are illustrated in order to give straightforward and in-depth physical explanations. Our results would be helpful for constructing future steerable optomechanical devices with more flexibility.

## 2. Numerical Simulation Model 

**Figure 1:**
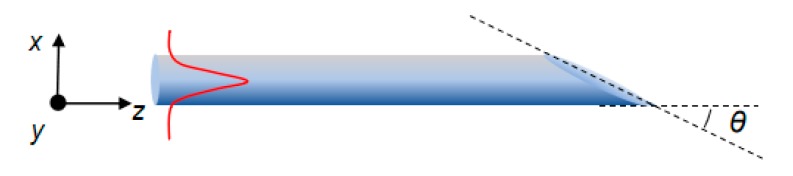
Schematic diagram of simulated subwavelength-diameter (SD)optical fibers with an oblique endface.

Figure 1 shows the schematic diagram of simulated SD optical fiber. An oblique cut endface, with an angle *θ* relative to fiber axis, was set at the output end of the optical fiber since such a structure would produce transverse optical force [35,36]. Cartesian coordinates were used here, where the fiber axis lies along the *z*-axis and cross sections overlap with the *x*-*y* plane. Circular cross sections of SD fibers were assumed. The material of the fiber was supposed to be silica (refractive index 1.45), which is linear and transparent in the near-infrared spectral range. The surroundings of the SD fiber were assumed to be a vacuum. A continuous-wave 980 nm light source of fundamental mode (i.e., HE_11_ mode) was excited and propagates along the fiber from left to right. Quasi-*x*, quasi-*y*, and associated circular (or elliptical) polarized fundamental modes were all simulated here. For numerical 3D-FDTD simulations, the simulation region was meshed with a cell size of 5 nm and terminated by a perfect matching layer boundary condition.

## 3. Results and Analyses

To calculate the optical force density inside SD fibers, the Einstein–Laub formula [31,35,36] was applied here to calculate the optical force density distributions together with three-dimensional FDTD simulations:

(1) $$F_{i}=1/2Real[(P\cdot \nabla )E^{*}+\left(\partial P/\partial t\right)\times \mu_{0}H^{*}]/\left(Input\;Power\right)$$

where $F_{i}$ denotes the time-averaged force density per unit volume (normalized to input light power); ***E*** and ***H*** represent the electric and magnetic fields, with superscript asterisk * denoting the complex conjugate; $P=\varepsilon_{0}\left(\varepsilon -1\right)E$ represents electric polarization density within the fiber, while ε represents the dielectric constant of silica with $\varepsilon_{0}$ and $\mu_{0}$ denoting vacuum permittivity and permeability, respectively.

**Figure 2:**
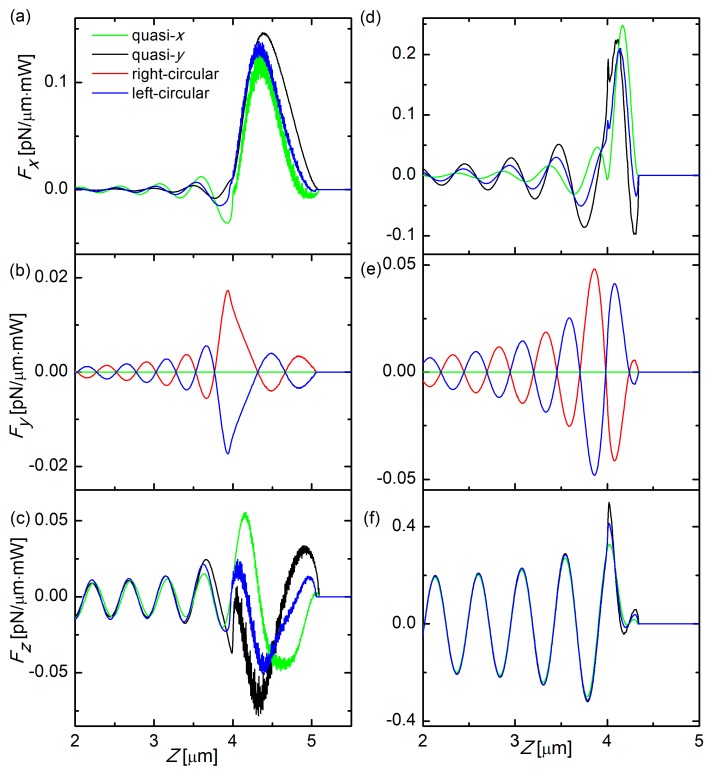
Calculated optical force components (integrated over transverse cross sections) are shown as a function of z upon quasi-linear and circular polarized excitations, (**a**) ***F_x_***, (**b**) ***F_y_***, (**c**) ***F_z_***, for an oblique (*θ* = 20°) endface; and (**d**) ***F_x_***, (**e**) ***F_y_***, (**f**) ***F_z_***, for an oblique (*θ* = 50°) endface. The labels in (**a**) apply to all other figures.

First, we investigated the polarization-dependent optical force inside SD fibers with oblique endfaces. As for the simulated wavelength at 980 nm, diameters of SD fibers were set to be 400 nm so as to ensure single mode waveguiding. As is known, fundamental modes, namely HE_11_, inside SD fibers, can be treated as quasi-linearly polarized (i.e., quasi-*x* or *y*) [37,38]. Additionally, circular (i.e., left or right circular) polarizations of fundamental modes can also be excited by simultaneously launching one quasi-*x* and one quasi-*y* polarized HE_11_ modes with the same magnitude but relative phase difference of π/2. In our simulations, when the quasi-y polarized HE_11_ mode was advanced in the phase of π/2 with respect to the quasi-*x* polarized HE_11_ mode, we then had excitation of the left-circularly polarized light mode; when the quasi-*x* polarized HE_11_ mode was advanced in the phase of π/2 with respect to the quasi-*y* polarized HE_11_ mode, we then had excitation of the right-circularly polarized light mode; for phase retardation other than π/2, the excited light mode was then elliptically polarized. Specifically, the length of simulated SD fiber was set to be 10 μm and the position of light source was set to be at the left end of the SD fiber, as schematically shown in Figure 1. The positions where SD fibers start to be oblique cut were kept as *z* = 4 μm. With input light of different polarizations, calculated optical force components (integrated over transverse cross sections) are shown as a function of *z* in Figure 2a–c for an oblique (*θ* = 20°) endface. Distributions of ***F_x_***, ***F_y_***, and ***F_z_*** all possess strong oscillations in the regions close to the fiber endface, which was attributed to quick radiation of reflected light fields from the oblique endfaces [35]. For ***F_x_*** and ***F_z_***, calculated force densities of all polarizations behave identically; especially for the right- and left-circular polarizations, there was no difference between the two which is reasonable considering the symmetry of simulated structure with respect to the circular polarizations of incident light modes. However, while ***F_y_*** for quasi-linear polarizations remain vanishing, it is noted that ***F_y_*** for circular polarizations exhibit non-zero values, as depicted in Figure 2b, even though the simulated geometries are symmetric with respect to the *x*-*z* plane. In addition, left- and right-circular polarized modes oscillated in a similar but opposite manner, as shown in Figure 2b. For right-circular polarization, the total ***F_y_*** integrated along the *z*-axis gave considerable magnitude, 0.0042 pN/mW, comparable to the total ***F_x_*** (0.047 pN/mW) and ***F_z_*** (−0.0302 pN/mW) for the quasi-*x* polarized mode. To understand this point, we have to refer to the fact that any interface for light and photons, for instance oblique fiber endfaces in this paper, all the photon-associated reflection, refraction, and diffraction are polarization-sensitive. As stated in the preceding section, circular polarizations can be treated as combinations of two orthogonal quasi-linear polarized modes with π/2 phase differences. During the waveguiding process, these two orthogonal modes will interfere with each other, together contributing to the actuation force exerted on the SD fiber endface. While encountering the oblique endface, reflection, refraction, and diffraction of the composite orthogonal modes would certainly behave differently, not to mention the phase difference therein. Therefore, at the endface of the SD fiber, circular polarized modes would suffer asymmetric reflections, radiations, and refractions, which finally results in unbalanced optical force in the *y*-direction. For comparison, we also performed investigations on a different endface with an inclination angle (*θ* = 50°), as shown in Figure 2d–f, displaying all the force density components with larger magnitudes, which is possibly related to the enlarged enface reflectivity with increasing inclination angle [35].

**Figure 3:**
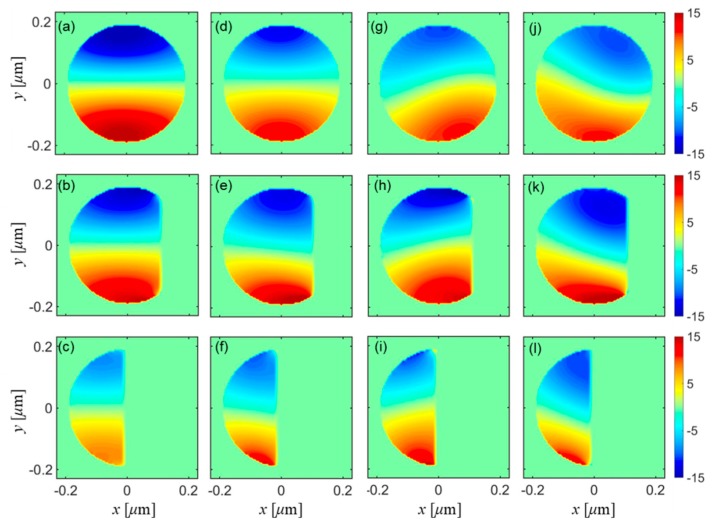
Transverse cross-sectional plots of the optical force-density component ***F_y_*** with varying distances to the oblique fiber endface (*θ* = 50°) for different polarizations: the left column corresponds to quasi-*x* polarized excitation in the central *x-y* plane with (**a**) *z* = 4 μm, (**b**) *z* = 4.1 μm, (**c**) *z* = 4.2 μm; the second column corresponds to right-circular polarized excitation in the central *x-y* plane with (**d**) *z* = 4 μm, (**e**) *z* = 4.1 μm, (**f**) *z* = 4.2 μm; the third column corresponds to elliptical polarized excitation with phase retardation of π/4 in the central *x-y* plane with (**g**) *z* = 4 μm, (**h**) *z* = 4.1 μm, (**i**) *z* = 4.2 μm; the fourth column corresponds to elliptical polarized excitation with phase retardation of 3π/4 in the central *xy* plane with (**j**) z = 4 μm, (**k**) z = 4.1 μm, (**l**) z = 4.2 μm. The color scale bar shows the force density in units of N/(m^3^·nW).

**Figure A1:**
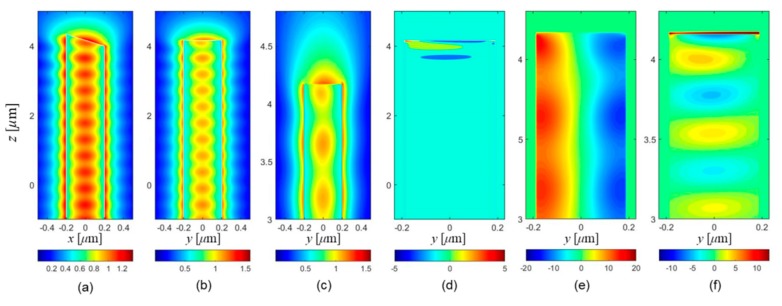
For an oblique endface (θ = 50°) by right-circular polarized excitations, transverse cross-sectional plots of optical fields in (**a**) *x-z* plane (*y* = 0 μm), (**b**) *y-z* plane (*x* = 0 μm), (**c**) enlarged *y-z* plane (*x* = 0 μm) and corresponding optical force density (**d**) ***F_x_***, (**e**) ***F_y_***, (**f**) ***F_z_*** in the *y-z* plane (*x* = 0 μm).

To better understand the origin of lateral (especially *y*-directional) optical force of circular polarized modes, detailed optical force density distributions were presented in this section. Specific parameters for simulations remain the same as in the above section, while the inclination angle *θ* is set to be 50°. Figure 3 shows the transverse cross-sectional plots of the optical force-density component ***F_y_*** with varying distances to the fiber endface for different polarizations. The left column of Figure 3 corresponds to quasi-*x* polarized excitation, while the second column corresponds to right-circular polarized excitation. The optical force-density components located on the outer boundary of fibers were removed so as to make the inner optical force density distributions more evident. From the left columns, it is easy to tell that distributions of ***F_y_*** were mirror-symmetric, and total vanishing *y*-directional optical force was found for quasi-*x* polarizations. While for the right-circular polarization, it is straightforward to note that distributions of ***F_y_*** were no longer mirror-symmetric in the *y*-direction, resulting in a non-vanishing *y*-directional optical force. Further investigations also demonstrate that cross-sectional distributions and radiations of electric fields, as shown in Figure A1, deviated from mirror-symmetry in the *y*-direction. These results are unusual considering that the oblique enface of the SD fiber is perpendicular with the *x-z* plane and thus is symmetric with respect to the *x-z* plane. For quasi-*x* or y linear polarization, it is reasonable to have mirror-symmetrically distributed ***F_y_***, and no net y-directional optical force was expected either. It is known that circular polarizations can be regarded as rotating polarized excitations along the fiber axis. Considering the fiber endface that terminates the optical fiber, where the cross section of the endface continuously shrinks towards the end of the fiber, such rotating polarization would thereafter experience a distinct light reflection, refraction, and diffraction at different positions of the fiber endface. This point can be clearly seen by checking the distribution of the optical field in the Appendix A Figure A1. Figure A1a–c displays asymmetric distributions and radiations of optical fields in the *x-z* plane and *y-z* plane, leading to asymmetric distributions of optical force densities as shown in Figure A1d–f. Accordingly, asymmetric distribution of ***F****_y_* produces net y-directional optical forces. The *y*-directional lateral optical force, only arising for circular-polarization upon which that asymmetric behavior occurs, is for the first time revealed here and provides more flexibility for actuating SD optical fiber based optomechanical devices.

**Figure 4:**
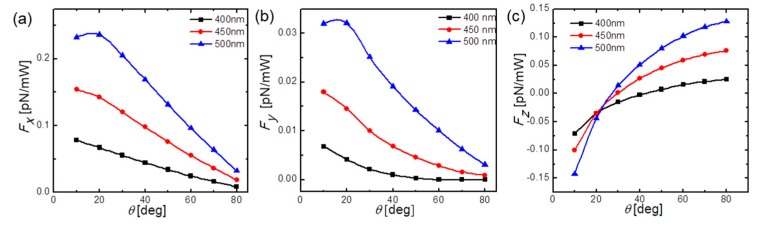
Calculated total optical force (**a**) ***F****_x_*, (**b**) ***F****_y_*, (**c**) ***F****_z_* upon varying inclination angles *θ* for SD optical fiber diameters of 400, 450, and 500 nm.

Furthermore, we also investigated the lateral optical force induced by elliptical polarized modes. As stated before, for phase retardations other than π/2 between the composite orthogonal quasi-linear polarized modes, we could have excitation of elliptical polarization. Here, phase retardations of π/4 and 3π/4, and an inclination angle *θ* of 50° were chosen for conducting simulations. The third and fourth columns of Figure 4 show the transverse cross-sectional plots of the optical force-density component ***F_y_*** with varying distances to the fiber endface for elliptical polarizations with phase retardations of π/4 and 3π/4, respectively. It is easy to find that for elliptical polarizations, the asymmetry of ***F_y_*** becomes more distinct compared with the circular-polarized input, while the sign of overall lateral optical forces, the same as shown in Figure 2b, is strongly related to the magnitude of phase retardations. It can be inferred that for elliptical polarizations, all the associated polarization-dependent reflection, refraction, and diffraction become more complicated and alternate significantly, which would in return contribute to more distinct *y*-directional non-zero optical forces. Besides, it was also found that an increase of SD fiber diameters could also lead to enlargement of lateral optical force, as shown in Figure 4. Indeed, we found that for either a decrease of oblique angle or an increase of fiber diameter, lateral optical forces, i.e., ***F_x_*** and ***F_y_***, were both enhanced. Considering the larger diameters and sharper inclination angles, the reflected light significantly deviated from the steady waveguiding modes and thus decayed much faster in a more asymmetric manner, so as to produce more distinct *x*- or *y*-directional lateral optical forces and associated lateral motion of SD optical fibers. It was also noted that ***F****_x_* is nearly one order magnitude larger than ***F_y_***, which is due to much more asymmetric distribution and radiation of optical fields as shown in Figure A1a–c. Different from ***F****_x_* and ***F_y_***, ***F_z_*** is generally increasing with a larger inclination angle. This point is reasonable considering that for flatter endfaces, more photons would exchange momentum in the longitudinal rather than lateral direction, contributing to a larger magnitude of *z*-directional optical forces. Overall, it is expected that lateral motions of the SD fiber endface would become more significant as long as optimal polarization and geometry are chosen. Benefitting from the polarization-dependent lateral optical forces obtained here, it can be easily concluded that the direction of lateral motion could also be specifically manipulated aside from the magnitude of lateral motions.

## 4. Conclusions

In conclusion, we have successfully investigated the force and force density distributions inside SD optical fibers under different polarized excitations. Our simulation results readily show that the input light of circular or elliptical polarizations would bring about lateral (i.e., *y*-directional) optical force, only arising for circular-polarization, which has not been observed in previous studies. By means of 3D-FDTD simulations, detailed profiles of the asymmetric transverse force density distributions were presented, which gave straightforward indications of the origin of newly found transverse optical force. Such symmetry breaking of optical force distributions was due to polarization-sensitive reflection, refraction, and diffraction of different electric fields occurring at the interface, i.e., fiber oblique endfaces here. Additionally, the enhancement of *y*-directional optical force was from zero for quasi-linear polarized excitation to 0.0042 pN/mW for circular (elliptical) polarized excitation. This significant enhancement of lateral *y*-directional optical force provides more flexibility for actuating SD optical fiber movements in multi-dimensions. Further study could reveal that such transverse optical force could be enhanced by elliptical polarizations or decreasing inclination angles. We believe our new findings could be helpful for constructing future steerable optomechanical devices with more flexibility.
